# The Ability of Quantitative, Specific, and Sensitive Point-of-Care/Chair-Side Oral Fluid Immunotests for aMMP-8 to Detect Periodontal and Peri-Implant Diseases

**DOI:** 10.1155/2018/1306396

**Published:** 2018-08-05

**Authors:** Saeed Alassiri, Pirjo Parnanen, Nilminie Rathnayake, Gunnar Johannsen, Anna-Maria Heikkinen, Richard Lazzara, Peter van der Schoor, Jan Gerrit van der Schoor, Taina Tervahartiala, Dirk Gieselmann, Timo Sorsa

**Affiliations:** ^1^Department of Oral and Maxillofacial Diseases, Helsinki University Hospital, University of Helsinki, Helsinki, Finland; ^2^Department of Dental Medicine, Division of Periodontology, Karolinska Institutet, Stockholm, Sweden; ^3^Institute of Molecular Dentistry, Solingen, Germany

## Abstract

The analysis of the disease-specific oral and systemic biomarkers in saliva and oral fluids (i.e., mouth rinse, gingival crevicular fluid (GCF), and peri-implantitis fluid (PISF)) is demanding. Several hosts and microbial factors may influence their expression, release, and levels. The type of saliva/oral fluids utilized for the diagnostics affects the analysis. High sensitivity and specificities together with sophisticated methods and techniques are essential for valuable outcome. We describe here recently developed practical, convenient, inexpensive, noninvasive, and quantitative mouth rinse and PISF/GCF/chair-side/point-of-care (PoC) lateral-flow aMMP-8 immunoassays (PerioSafe and ImplantSafe/ORALyser) to detect, predict, and monitor successfully the course, treatment, and prevention of periodontitis and peri-implantitis, respectively. The tests have been independently and successfully validated to differentiate periodontal and peri-implant health and disease in Finland, Germany, Netherland, Sweden, Turkey, Nigeria, Malawi, and USA. The clinical use of salivary/oral fluid biomarkers to identify oral and systemic conditions requires additional studies utilizing these noninvasive screening, diagnostic, and preventive aMMP-8 PoC/chair-side technologies.

## 1. Introduction

Diagnosis of periodontal and peri-implant diseases are mostly based on an array of clinical measurements and indices including pocket probing depths, bleeding on probing, and assessment of clinical attachment level together with radiographic findings. Additional information such as medical, hereditary, and specific features and the amount of dental plaque have also been recorded [1, 2]. These laborous diagnostic procedures require not only multiple manual recordings but also professional examiners with trained expertise. So far, these clinical and radiographic diagnostic procedures are the best currently available ones for diagnosing and monitoring the disease course, treatment, and maintenance [1]. However, they can only assess the past experience, current extent, and severity of the periodontal and peri-implant diseases. Since these clinical and radiographic analyses of periodontitis and peri-implantitis have low sensitivity and low positive predictive value [1], no reliable information can be obtained regarding the diseases' current activities and their future courses [1]. Also, the episodic progression of periodontitis and peri-implantitis makes the accurate assessment of disease activity and progression difficult and complicated [1]. Neutrophil collagenase, also called matrix metalloproteinase (MMP)-8, polymorphonuclear (PMN) leukocyte collagenase, or collagenase-2, has been identified and characterized as a major collagenolytic enzyme that causes active periodontal and peri-implant degeneration (APD) in periodontitis and peri-implantitis [3–7]. MMP-8 can resolve and regulate inflammatory and immunological cascades by processing nonmatrix bioactive substrates such as chemokines, cytokines, serpins, and complement components. Physiological levels of MMP-8 can exert protective and defensive anti-inflammatory characteristics [5]. Increased levels of especially active MMP-8 (aMMP-8), but not latent, inactive proform, have been found in periodontitis- and peri-implantitis-affected oral fluids (saliva, mouth rinse, gingival crevicular fluid (GCF), and peri-implant sulcular fluid (PISF)) [8–10]. A key characteristic of active periodontal and peri-implant diseases is the sustained pathological elevation and activation of MMP-8 in periodontal and peri-implant tissues, which are reflected in oral fluids [4]. Consequently, aMMP-8 is a promising biomarker candidate for diagnosing and assessing the progression and course of these episodic oral inflammatory tissue destructive and degenerative diseases [3, 4]. More importantly, aMMP-8 in oral fluids can also serve as a predictive and preventive adjunctive biotechnological tool to indicate [4, 7, 8, 11] and time the preventive interventions (secondary prevention or supportive periodontal/peri-implant therapy [12, 13]) and to inhibit or reduce the conversion of preperiodontitis (formerly gingivitis) and preperi-implantitis (formerly mucositis) to periodontitis and implantitis, respectively.

With this background, we describe the documentation of currently commercially available quantitative reader-based aMMP-8 oral fluid specific point-of-care/chair-side lateral-flow reader-equipped immunotests, that is, PerioSafe and ImplantSafe/ORALyser, for periodontal and peri-implant diseases, respectively.

## 2. PerioSafe and ImplantSafe, aMMP-8 Oral Fluid PoC/Chair-Side Tests, in Chronic Periodontitis and Peri-Implantitis: Diagnostic Utilizations and Monitoring the Effects of Treatments

Recently, lateral-flow point-of-care (PoC)/chair-side tests (PerioSafe and ImplantSafe), discovered in Finland and further developed in Germany [4, 14], have been developed based on earlier described technologies and monoclonal antibodies [4, 5, 14, 15]. The tests, PerioSafe and ImplantSafe, and reader (ORALyser) have been developed and manufactured by Medix Biochemica Ltd (Espoo, Finland) and dentognostics GmbH (Jena, Germany) and are commercially available from Dentognostics GmbH (Jena, Germany). In fact, the PoC/chair-side aMMP-8 lateral-flow immunotests resemble the classical pregnancy and/or recently described HIV-PoC tests [16, 17]. The aMMP-8 oral fluid tests can be used according to the manufacturer's instructions [5]. PerioSafe measures and analyses the levels of aMMP-8 in mouth rinse and ImplantSafe in PISF and GCF; thus, PerioSafe is patient-specific and ImplantSafe is site-specific [5, 14]. PerioSafe and ImplantSafe test-sticks can be quantitated by the ORALyser reader in 5 min PoC/chair-side [4]. PerioSafe and ImplantSafe with ORALyser quantitation are reliable, quantitative, noninvasive, safe, and inexpensive adjunctive point-of-care diagnostic tools for diagnosis, screening, monitoring, and prevention of periodontal and peri-implant diseases [4, 5].

**Figure 1:**
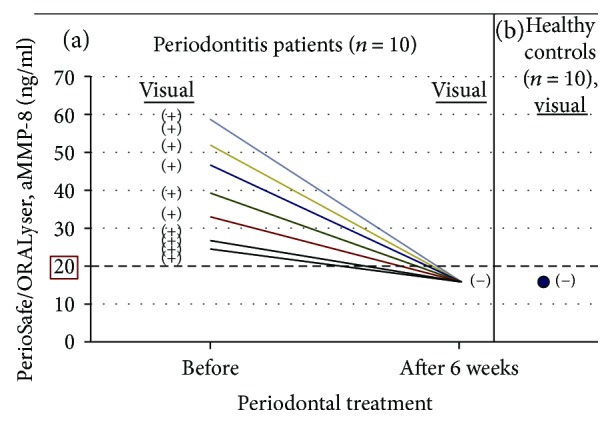
aMMP-8 chair-side levels analysed visually (+/−) and quantitated for aMMP-8 (ng/ml) by ORALyser reader to monitor the effect of periodontal treatment (scaling and root planing) on chronic periodontitis (*n* = 10) (a) and healthy controls (*n* = 10) (b).

Chronic periodontitis (*n* = 10), peri-implantitis (*n* = 30), and their healthy controls (*n* = 10 and 30, resp.) were characterized clinically and from X-rays as described earlier [11, 15, 18, 19]. Ten clinically and X-ray-diagnosed adult chronic periodontitis patients [11, 15] were all (100%) diagnosed to be aMMP-8 positive by PerioSafe visual test, and their aMMP-8 lateral-flow test-sticks were quantitated by ORALyser before (all >20 ng/ml, visually (+)) and after periodontal treatment, scaling and root planing (SRP) or anti-infective treatment (Figures 1(a) and (b)). SRP or anti-infective treatment was found to reduce the pocket depths and the bleeding of probing [11, 15]. SRP also affected the aMMP-8 levels in mouth rinse from positive (+) to negative (−) by visual estimation of the test results and from >20 ng/ml (positive (+)) to <20 ng/ml (negative (−)) by ORALyser reader quantitation (Figure 1(a)). Systemically and periodontally healthy dental students (age 22–24, *n* = 10) served as healthy controls (Figure 1(b)).

**Figure 2:**
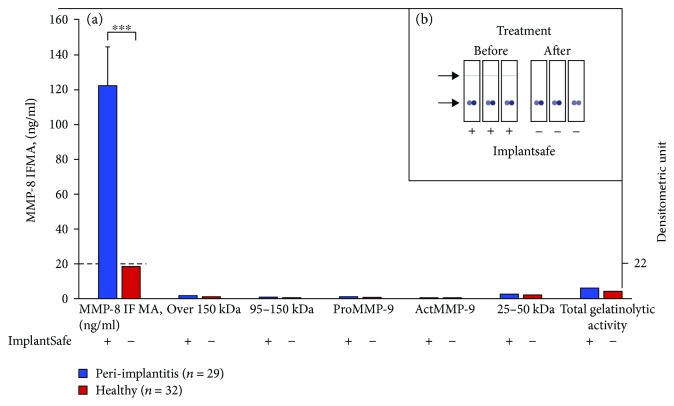
Peri-implant sulcular fluid (PISF) collected from 29 peri-implantitis and 32 periodontally healthy sites were tested for elevated aMMP-8 by ImplantSafe PoC/chair-side test (+/−), analysed for aMMP-8 (ng/ml) by immunofluorometric assay (IFMA), and by quantitated gelatin zymography for all molecular weight forms of gelatinase-B (MMP-9, zymographic densitometric units) (a). Implantitis sites were tested before and after surgical treatment by ImplantSafe (+/−); treatment caused positive sites to be negative (b). Elevated aMMP-8 levels in PISF detected by ImplantSafe positivity and IFMA associated significantly with peri-implantitis (*p* < 0.0001, Wilcoxon test (^∗∗∗^)) and could thus be conveniently PoC detected in 5 min by ImplantSafe. Any forms or total MMP-9 did –not differentiate peri-implantitis and healthy sites (a).

We further demonstrated here that the ImplantSafe aMMP-8 PoC/chair-side sulcular fluid test site specifically in 5 min detects the peri-implantitis sites (*n* = 29) differentiating them from clinically healthy peri-implant sites (*n* = 32) and can be utilized for monitoring treatment (Figures 2(a) and 2(b)). Peri-implantitis and healthy sites were diagnosed clinically and by X-rays as described [18]. Peri-implantitis sites were surgically treated according to the Swedish national guidelines [19]. We also analysed quantitatively aMMP-8 by immunofluorometric assay (IFMA) [15] and all forms of MMP-9 densitometrically by quantitated gelatin-zymography [20]. Similar to ImplantSafe PoC/chair-side findings, elevated aMMP-8 levels could be detected by IFMA in all peri-implantitis sites (29 = 100% ImplantSafe-positive >20 ng/ml (124,60  ± 22.50 ng/ml)) differing from clinically healthy sites all having low aMMP-8 levels (32 sites/<20 ng/ml (18.60 ± 3.46 ng/ml)) all being ImplantSafe PoC-negative (Figure 2(b)). This difference was statistically significant (*p* < 0.0001, Wilcoxon test) (Figure 2(a)). Surgical treatment of peri-implantitis sites according to the Swedish national guidelines [19] caused the positive ImplantSafe tests to be negative (Figure 2(b)). This pilot case-control peri-implantitis study shows both 100% sensitivity and specificity for ImplantSafe test. Currently, this test is available also as a reader-equipped/quantitated-PoC tool, that is, ORALyser reader [4]. MMP-9 or gelatinase-B, analysis by gelatin zymography from these same PISF samples, revealed that any form of MMP-9 or total MMP-9 was not able to differentiate peri-implant health and disease (Figure 2(a)). Our pilot case-control studies have received approval from the local ethical committee of Stockholm Community, Sweden (2016-08-24/2016/1:8 and 2016-1-24) and the Helsinki University Central Hospital, Finland (nro260/13/03/00/13).

## 3. Discussion

Currently, the practical and quantitative PoC/chair-side lateral-flow oral fluid aMMP-8 immunoassays have been successfully developed and are commercially available. The tests have been independently and successfully validated in Finland, Nigeria, Germany, Holland, Malawi, Turkey, Sweden, and USA [4, 5, 14, 21–24]. The tests have diagnostic sensitivity and specificity 76–90% and 96%, respectively, corresponding to odds ratio of >72 [4, 5, 14, 21]. The test results are quantitatively available by the reader in 5 min PoC/chair-side [4]. The tests have been shown to be useful to screen susceptible sites and patients, differentiate active and inactive periodontitis and peri-implantitis sites, predict the future disease progression, and monitor the treatment—with or without different adjunctive medications—response, and maintenance therapy [4, 5]. As demonstrated in the present study, the tests excellently differentiated periodontal and peri-implant health and diseases. Additionally, the test can identify initial and alarm or early periodontitis (preperiodontitis) in genetically predisposed adolescents [21]. Thus, the test is effective in both adult and adolescent populations and can act in “a gene”-test manner [14, 21]. Pathologically elevated aMMP-8 associates, reflects, and precedes the active phase of periodontal and peri-implant diseases, that is, APD [4, 7, 8]. This forms the basis of predictive value for aMMP-8 oral fluid tests in periodontal and peri-implant diseases; thus, the test is positive ahead or before clinical manifestations and/or radiographic outcomes of APD [4, 5, 8]. The tests can detect enough early subclinical and silent developing preperiodontitis and preperi-implantitis [4, 21]. The tests are very suitable for monitoring the disease's development and progression as well as timing and targeting the preventive and therapeutic interventions (i.e., secondary prevention and/or supportive periodontal/peri-implant therapy) [12, 13]. In comparison to bleeding on probing, aMMP-8 PoC/chair-side test is much more sensitive to distinguish periodontal health and disease [14]. Noteworthy, bleeding on probing always causes bacteraemia. Noninvasive PerioSafe and ImplantSafe testings never cause bacteraemia.

Regarding other PoC technologies for periodontitis and peri-implantitis, Ritzer et al. [25] described peri-implantitis/periodontitis online diagnosis utilizing elegantly the tongue as a 24/7 detector allowing diagnosis by “anyone, anywhere, and anytime.” Their clever technology utilizes chewing gum-containing peptide sensors as protease cleavable linkers between a bitter taste substance and a microparticle. MMPs upregulated in oral cavity and fluids (i.e., saliva and GCF/PISF) are presented to be responsible for cleaving the sensor in chewing gum generating the bitter taste consequently to be detected by the tongue as a 24/7 in vivo and online sensor [25]. Nonetheless, their elegant 24/7 tongue sensor assay is not MMP-specific for the main collagenolytic MMP, MMP-8, upregulated and activated in periodontitis- and peri-implantitis-affected periodontal and peri-implant tissue, gingiva and oral fluids, including saliva, GCF, PISF, and mouth rinse. The sensor is sensitive also to cleavages by MMP-1, MMP-13, and MMP-9 present and upregulated in the diseased periodontitis and peri-implantitis tissue and oral fluids [25]. Furthermore, no information was provided regarding specificity, sensitivity, and predictability of the 24/7 tongue technology regarding periodontitis and peri-implantitis. PerioSafe and ImplantSafe exert 76–90% specificity and >96% sensitivity [4, 5]. Also, the susceptibilities of the protease cleavable linker 24/7 sensor to catalytically competent and effective microbial proteases expressed and released by dysbiotic oral periodontopathogens and candidial species were not addressed [26, 27]. Recently, a PoC/chair-side device, comparable to PCR-detection, for *Porphyromonas gingivalis* related to chronic periodontitis detection has been developed [28]. A chair-side assay for GCF calprotectin has been described [29]. Calprotectin does not degrade anything [29].

PerioSafe and ImplantSafe are specific and sensitive for aMMP-8, a major destructive and collagenolytic factor for APD [4, 5]. Overall, PerioSafe and ImplantSafe, aMMP-8 quantitative oral fluid PoC/chair-side lateral-flow immunotests, are the first clinically validated commercially available diagnostic, prognostic, quantitative, predictive, noninvasive, and preventive PoC/chair-side technologies for periodontal and peri-implant diseases [4, 5]; the tests can be used by dental and medical professionals linking these disciplines [4]. Potentially, such aMMP-8 PoC tests can eventually be adapted for other medical disciplines [30, 31].
